# Whole exome sequencing of urachal adenocarcinoma reveals recurrent NF1 mutations

**DOI:** 10.18632/oncotarget.8640

**Published:** 2016-04-07

**Authors:** Harshabad Singh, Yang Liu, Xiuli Xiao, Ling Lin, Jaegil Kim, Paul Van Hummelen, Chin-Lee Wu, Adam J. Bass, Philip J. Saylor

**Affiliations:** ^1^ Massachusetts General Hospital Cancer Center, Boston, MA, USA; ^2^ Dana Farber Cancer Institute, Boston, MA, USA; ^3^ Department of Pathology, Massachusetts General Hospital, Boston, MA, USA; ^4^ Broad Institute, Cambridge, MA, USA

**Keywords:** urachal adenocarcinoma, NF1, whole exome sequencing

## Abstract

Urachal adenocarcinoma is a rare bladder malignancy arising from the urachal remnant. Given its rarity and the lack of knowledge about its genetic characteristics, optimal management of this cancer is not well defined. Practice patterns vary and outcomes remain poor. In order to identify the genomic underpinnings of this malignancy, we performed whole exome sequencing using seven tumor/normal pairs of formalin fixed archival specimens. We identified recurrent evidence of MAP-kinase pathway activation as three patients had neurofibromin 1 (*NF1*) mutations, with one of these patients also harboring an oncogenic *KRAS* G13D mutation. We also observed recurrent evidence of Wnt/β-catenin pathway activation as three patients had oncogenic mutations in *APC* or *RNF43*. In addition, somatic copy number analysis revealed focal chromosome 12p amplifications in three samples, resembling findings from testicular germ cell tumors. We describe the genomic landscape of this malignancy in our institutional cohort and propose investigation of the therapeutic potential for MAP-K pathway inhibition in the subset of patients who show evidence of its activation.

## INTRODUCTION

The urachus, a fibrous allantoic remnant connecting the bladder to the umbilical cord during embryogenesis, typically regresses after birth to form the median urachal ligament. However, the urachal remnant may persist in up to one third of people. Urachal adenocarcinoma is a rare malignancy arising in this urachal remnant and represents <1% of all bladder cancers. In contrast to typical bladder cancers that have a transitional cell histology, urachal tumors are typically mucinous adenocarcinomas and often display focal signet ring features.[1, 2] These tumors often present at an advanced stage and have an overall survival of 40-50% at 5 years. [2, 3]

Clinical care of patients with urachal adenocarcinoma is limited by a dearth of clinical trials and by a lack of detailed insights into the pathophysiology of the disease. Importantly, knowledge of the underlying somatic genomic alterations in urachal adenocarcinoma is limited. Focused genomic studies examining specific ‘hotspot’ DNA mutations identified recurrent *KRAS* mutations.[4] A recent whole exome sequencing effort to characterize cancers afflicting adolescents and young adults included one patient with urachal carcinoma and also identified an activating KRAS mutation. To our knowledge, there has been no unbiased systematic effort to describe genomic alterations in urachal adenocarcinomas. [5]

We therefore performed whole exome sequencing (WES) of tumor and matched germline DNA from seven patients with urachal adenocarcinoma in an effort to discover commonly altered pathways and identify potential targets for therapy.

## RESULTS

Formalin-fixed archival samples of urachal adenocarcinoma from eight patients were obtained from within the institutional cohort at Massachusetts General Hospital. Patient demographics are listed in Table 1. From these samples, DNA was isolated from regions of malignant and normal tissue and then subjected to WES. One tumor sample that was >20 years of age showed poor sequencing coverage (Supplemental Table 1) and was excluded from further analysis. From the remaining seven tumor/normal pairs, we achieved a mean coverage of 73x across target bases with 86% of targeted bases covered at 30x or greater. We evaluated the tumor DNA samples relative to the matched normal tissue DNA to identify somatic mutations, insertion/deletion events, and somatic copy-number alterations (SCNAs; algorithms detailed in Methods section). Across the coding genome, the mean mutation prevalence was 1.63 mutations/Mb (Range 0.82 – 2.53). (Supplementary Table 2)

**Table 1:** Patient demographics and survival data

Patient ID	Gender	Year of diagnosis	Age at diagnosis (Years)	Smoking history	Stage at diagnosis (Localized vs. Metastatic)	Initial Treatment	Alive or Dead (Disease status/Cause of death)	Relapse Free Survival (days)	Overall Survival (days)
2T	F	1988	51	Yes	Localized	Radical cystectomy, RT	Dead (Ds recurrence)	1460	2912
3T	F	2000	82	NA	Localized	Radical cystectomy	Dead (Ds recurrence)	397	716
4T	M	2005	45	Yes	Localized	Partial cystectomy, RT	Alive (NED)	3665	3665
5T	F	2009	45	NA	Localized	Partial cystectomy, RT	Dead (Ds recurrence)	348	455
6T	M	2011	20	Yes	Localized	Partial cystectomy, Adjuvant ITP chemotherapy	Alive (NED)	1613	1613
7T	M	2013	62	Yes	Localized	Partial cystectomy	Alive (NED)	809	809
8T	F	2014	52	Yes	Metastatic	Debulking surgery, FOLFIRI chemotherapy	Alive (Progressive ds)	NA	578

RT – Radiation therapy, ITP – Ifosfamide (I), Paclitaxel (T), Cisplatin (P), FOLFIRI – 5-Fluorouracil, Leucovorin, Irinotecan; NA – Not available, ds recurrence – disease recurrence, NED – No evidence of disease.

**Figure 1:**
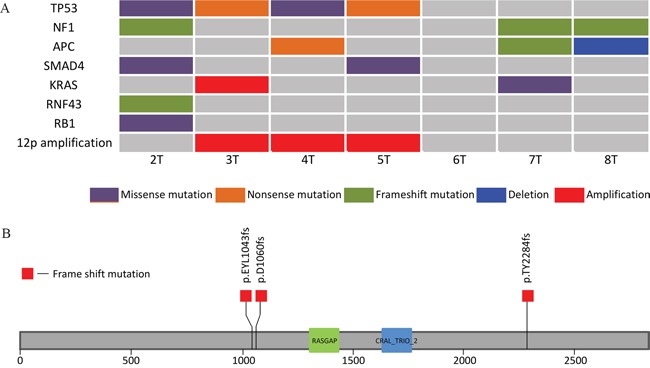
**A.** Comutation plot for the 7 samples. **B.** Neurofibromin 1 gene with its two protein domains showing the location of the three frame shift mutations identified in our cohort. The RASGAP domain (Ras-like GTPase) lies between amino acids 1342-1451 and the CRAL_TRIO_2 domain lies between amino acids 1602 – 1736.

We next evaluated candidate pathogenic mutations. Using MutSigCV, we identified three genes with recurrent mutations: *TP53, NF1* and *SMAD4* (Figure 1A). *TP53* mutations were found in four of the seven patients in our cohort. Two patients had a nonsense mutation (R65*, R342*) and the other two had missense mutations (N239D, R248Q) described previously in other malignancies. [6] Most strikingly, three of the seven patients harbored *NF1* frame-shift events that would be expected to inactivate this tumor suppressor (Figure 1B). Two samples contained *SMAD4* R361C missense mutations, the most common *SMAD4* mutations in the COSMIC database.

To supplement this list of genes, we queried our sample set for specific mutations that have been seen recurrently in other cancers and are likely pathogenic drivers. This analysis revealed two samples with loss of function mutations in *APC*. An additional sample possessed frame-shift mutation in *RNF43*, an additional negative regulator of Wnt signaling. One sample harbored an inactivating *RB1* mutation. We also identified a single sample with a G13D *KRAS* mutation, intriguingly in a tumor also harboring an *NF1* frame-shift mutation.

**Figure 2:**
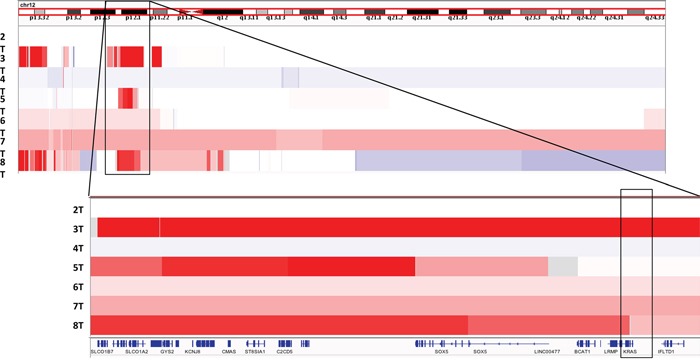
View of recurrent chromosome 12p copy number gains in our sample cohort viewed using Integrated Genome Viewer (IGV)[11] Amplified region and included genes among samples 3T, 5T, and 8T shown and highlighted.

We next evaluated the SCNAs in our sample set. Most strikingly, we found that three cases possessed focal amplifications within chromosome 12p12.1 (Figure 2). Interestingly, all three cases with 12p amplification also carried a *TP53* mutation. The amplified segment contained *KRAS* in sample 3T but this gene was not included in the amplicon in the two other samples. The minimal common amplified regions in the three samples contained 8 genes – *GYS2, LDHB, KCNJ8, ABCC9, CMAS, ST8SIA1, C2CD5, ETNK1*, making the etiology of these recurrent events still unclear. We also identified deletion on chromosome 5 in three samples (4T, 7T, 8T), which spanned over 20Mb and involved the *APC* gene, which was mutated in two other samples (5T, and 7T). One sample (4T) had a focal deep deletion of tumor suppressor *CDKN2A* (p16). We noted no clear focal amplifications at the loci of genes encoding receptor tyrosine kinases that are subject to therapeutic blockade (e.g. *MET* or *ERBB2*).

## DISCUSSION

To our knowledge, this work represents the first systematic unbiased sequencing effort to attempt to understand the genomic underpinnings of urachal adenocarcinomas. We identify recurrent loss of function *NF1* mutations. NF1 is a Ras-GTPase activating protein (GAP) and its loss is associated with increased formation of Ras-GTP leading to increased downstream signaling.[7] Somatic *NF1* mutations have been identified as pathogenic events in other malignancies like juvenile myelomonocytic leukemia, lung cancer and melanoma. Given its role in activating the RAS pathway, there has been interest in utilizing blockade of MEK and other members of the MAP-Kinase pathway in these cancers, a paradigm that may merit further study in urachal adenocarcinoma.

The functional significance of focal recurrent amplifications of chromosome 12p is not clear, however this amplified region did include *KRAS* in one of the three patients. The minimal shared amplified segment does not contain any clear oncogenes. It is also interesting to note that chromosome 12p is recurrently amplified in testicular germ cell tumors, another embryonal tumor. No clear oncogenic driver in this location has been identified.[8–10]

Prior genetic analysis of this disease has been limited. Sirintrapun et al. described a series of 7 patients with urachal adenocarcinoma and profiled the tumors for KRAS, BRAF hotspot mutations, and microsatellite instability. They identified recurrent KRAS mutations in 3 patients.[4] However, given the focused genomic analysis other genes we found to be recurrently mutated including NF1, TP53 were not evaluated. Cha et al. recently conducted a WES series of cancers afflicting adolescents and young adults which featured only one patient with urachal carcinoma carrying a KRAS G13D mutation.[5] Both studies underscore the importance of the RAS/MAPK pathway in this disease, a finding that is prominently highlighted by our study demonstrating not only KRAS mutations but also recurrent NF1 mutations and KRAS amplifications as likely mechanisms of MAPK pathway activation.

Lastly, our results show evidence for Wnt pathway activation (*APC* mutation/deletion and *RNF43* mutation) and alterations of the TGF-β pathway (*SMAD4* mutation). This constellation of recurrent p53, WNT/TGF-β and MAP-K pathway alterations bears some thematic resemblance to the genomics of colorectal cancer.

In conclusion we have performed whole exome sequencing analysis on a set of seven urachal adenocarcinomas, a rare form of bladder tumor. We observed evidence of recurrent loss of *NF1* and frequent activating mutations in the MAPK pathway. These findings merit validation in larger sample sets and have relevance to the study of therapies targeting the MAPK pathway in this disease.

## SUPPLEMENTARY METHODS TABLES


