# Supplementary material for: Whole exome sequencing of urachal adenocarcinoma reveals recurrent NF1 mutations
Source: Oncotarget. 2016 Apr 7;7(20):29211–5. doi: 10.18632/oncotarget.8640 (PMC5045390; doi:10.18632/oncotarget.8640)
Supplement: Supplementary file 1 [file oncotarget-07-29211-s001.pdf]

Whole exome sequencing of urachal adenocarcinoma reveals recurrent NF1 mutations

SUPPLEMENTARY METHODS

Our study was approved by the institutional IRB (Dana Farber / Harvard Cancer Center protocol 15-025). Formalin fixed paraffin embedded (FFPE) samples were collected from tissue blocks either from surgical resection specimens or biopsies of patients with a diagnosis of urachal adenocarcinoma at Massachusetts General Hospital (MGH), Boston. Samples were selected based on pathologic diagnosis according to standard guidelines for urachal adenocarcinoma. [1, 2] All pathology specimens were reviewed and reported by staff physicians specializing in genitourinary pathology in the Department of Pathology at MGH. In addition, the clinical charts for these patients were reviewed by two of the authors (HS, and PS) to confirm the diagnosis in question. Selection for sequencing was limited to samples for which there was concordance between the pathology report and treating physicians' diagnosis. Samples were grossly macrodissected and areas of pure tumor and pure surrounding normal tissue were obtained by punch biopsy.

Whole exome sequencing

DNA was extracted from FFPE tissue using truXTRAC FFPE DNA kit (Covaris, Woburn, MA) and quantified using Picogreen (Invitrogen, Carlsbad, CA). Whole Exome sequencing was performed using the platforms at the Center for Cancer Genome Discovery (CCGD). DNA was fragmented to 250bp (Covaris Inc, Woburn, MA) and further purified using Agencourt AMPure XP beads. 50 ng size selected DNA was then ligated to specific adaptors with sample specific barcodes during library preparation using KAPA DNA library preparation kit (KAPA Biosystems, Inc,Woburn, MA). All libraries were pooled and sequenced on a Illumina Miseq (Illumina Inc, San Diego, CA) for a relative quantification, and then normalized based on the number of reads. Normalized libraries were again pooled and enriched for the Exome V5 spiked in with a customized translocation baitset using Agilent Sureselect Hybrid Capture kit (Whole Exome_v5 Agilent, Santa Clara, CA). Several captures were pooled further and sequenced over 5 lanes to an equivalent of 2 exomes per lane on a Hiseq 2500 (Illumina Inc, San Diego, CA). [3]

Pooled sample reads were de-convoluted (de-multiplexed) and sorted using the Picard tools (http://picard.sourceforge.net). Reads were aligned to the reference sequence b37 edition from the Human Genome Reference Consortium using bwa and duplicate reads were identified and removed using the Picard tools. Recalibration of the quality scores was performed using GATK tools. [4–6]

Mutation analysis for single nucleotide variants (SNV) was performed using MuTect v1.1.4 in paired mode using matching normal and annotated by Oncotator. [7,8] We used the SomaticIndelDetector tool that is part of the GATK for indel calling. Consecutive variants in the same codon were reannotated to maximize the effect on the codon and marked as "Phased" variants.

Copy number analysis

Copy number variants were identified using RobustCNV, an algorithm in development at the CCGD. RobustCNV relies on localized changes in the mapping depth of sequenced reads in order to identify changes in copy number at the loci sampled during targeted capture. This strategy includes a normalization step in which systematic bias in mapping depth is reduced or removed using robust regression to fit the observed tumor mapping depth against a panel of normals (PON) sampled with the same capture bait set. Observed values are then normalized against predicted values and expressed as log2 ratios. A second normalization step is then done to remove GC bias using a loose fit.

Normalized coverage data is next segmented using Circular Binary Segmentation [9] with the DNAcopy Bioconductor package. Finally segments are assigned gain, loss, or normal-copy calls using a cutoff derived from the within-segment standard deviation of post-normalized mapping depths and a tuning parameter which was set based on comparisons to array-CGH calls in separate validation experiments.

A final step includes a centering of the Log2Ratios on diploid chromosomes determined by the allele fraction of heterozygous SNPs in the targeted panel.

We then summarized segment calls to gene calls by assigning segment calls to capture intervals and tallying interval calls for each gene. Genes may contain multiple intervals with a combination of calls; therefore, a variety of gene calls are possible.

REFERENCES

1. Johnson DE, Hodge GB, Abdul-Karim FW, Ayala AG. Urachal carcinoma. Urology 1985;26:218–21.
2. Dhillon J, Liang Y, Kamat AM, Siefker-Radtke A, Dinney CP, Czerniak B, et al. Urachal carcinoma: a pathologic and clinical study of 46 cases. Hum Pathol 2015;46:1808–14. doi:10.1016/j.humpath.2015.07.021.
3. Brastianos PK, Carter SL, Santagata S, Cahill DP, Taylor-Weiner A, Jones RT, et al. Genomic Characterization of Brain Metastases Reveals Branched Evolution and Potential Therapeutic Targets. Cancer Discov 2015;5:1164–77. doi:10.1158/2159-8290.CD-15-0369.
4. McKenna A, Hanna M, Banks E, Sivachenko A, Cibulskis K, Kernytsky A, et al. The Genome Analysis Toolkit: a MapReduce framework for analyzing next-generation DNA sequencing data. Genome Res 2010;20:1297–303. doi:10.1101/gr.107524.110.
5. Li H, Durbin R. Fast and accurate short read alignment with Burrows-Wheeler transform. Bioinforma Oxf Engl 2009;25:1754–60. doi:10.1093/bioinformatics/btp324.
6. DePristo MA, Banks E, Poplin R, Garimella KV, Maguire JR, Hartl C, et al. A framework for variation discovery and genotyping using next-generation DNA sequencing data. Nat Genet 2011;43:491–8. doi:10.1038/ng.806.
7. Cibulskis K, Lawrence MS, Carter SL, Sivachenko A, Jaffe D, Sougnez C, et al. Sensitive detection of somatic point mutations in impure and heterogeneous cancer samples. Nat Biotechnol 2013;31:213–9. doi:10.1038/nbt.2514.
8. Ramos AH, Lichtenstein L, Gupta M, Lawrence MS, Pugh TJ, Saksena G, et al. Oncotator: cancer variant annotation tool. Hum Mutat 2015;36:E2423–9. doi:10.1002/humu.22771.
9. Olshen AB, Venkatraman ES, Lucito R, Wigler M. Circular binary segmentation for the analysis of array-based DNA copy number data. Biostat Oxf Engl 2004;5:557–72. doi:10.1093/biostatistics/kxh008.

Supplementary Table 1: Final sequencing metrics

Sample ID	Total Reads	% PF Reads	% Selected bases	Mean Exon Target Coverage	% Duplication	% Exon Target Bases 30x	% Exon Targets Not Covered
1T*	93301106	96.7021	31.6294	15.04	46.104	5.3603	1.0924
1N*	101078746	96.8635	42.3129	32.12	25.9561	46.1059	1.0247
2T	178189890	96.3916	40.1485	48.47	35.2284	70.4778	1.1028
2N	248021644	92.1085	45.4318	62.98	43.1122	85.8315	1.0568
3T	108674080	96.4285	40.6206	31.47	30.297	44.3211	1.1423
3N	301690918	91.7694	45.3904	77.45	29.905	93.7988	0.9904
4T	87949984	96.5934	67.1332	51.89	31.1905	72.1967	0.888
4N	77336754	96.5142	54.0829	39.48	22.3642	59.803	0.8927
5T2	96435924	96.9696	65.9052	57.47	31.6562	83.3645	0.9956
5N	113538702	96.6569	63.7302	59.55	35.6646	87.4932	0.9383
6T	145427274	96.6464	66.2296	85.49	30.7925	93.3	0.7742
6N	124199380	96.501	62.8213	69.62	28.5244	85.6172	0.7929
7T	128572554	96.1603	73.8838	100.46	18.4215	89.4702	0.7439
7N	84315072	96.4327	69.5232	61.79	17.6123	82.5528	0.812
8T	115369564	96.5238	71.8989	89.04	15.5005	88.8644	0.888
8N	67433010	96.6117	70.2513	52.77	15.0674	76.0098	0.9934
CEPH1328	102414248	96.5505	71.2206	76.37	10.9087	90.1682	0.7304

* - Samples were excluded because of poor sequencing coverage

Supplementary Table 2: Log of number of mutations across sample set

Sample ID	Non Silent coding mutations	Small Insertion Deletions	Mutation Rate
2T	168	27	2.53
3T	73	30	0.82
4T	80	15	1.23
5T2	50	0	1.02
6T	127	8	2.34
7T	95	14	1.62
8T	119	12	1.87
